# Influence of the training set on the accuracy of surface EMG classification in dynamic contractions for the control of multifunction prostheses

**DOI:** 10.1186/1743-0003-8-25

**Published:** 2011-05-09

**Authors:** Thomas Lorrain, Ning Jiang, Dario Farina

**Affiliations:** 1 Sensory-Motor Interaction, Department of Health Science and Technology, Aalborg University Denmark; 2 Department of Neurorehabilitation Engineering, Bernstein Center for Computational Neuroscience, University Medical Center Göttingen, Georg-August University, Göttingen, Germany; 3 Otto Bock HealthCare GmbH, Strategic Technology Management, Max-Näder-Str. 15, D-37115 Duderstadt, Germany

## Abstract

**Background:**

For high usability, myo-controlled devices require robust classification schemes during dynamic contractions. Therefore, this study investigates the impact of the training data set in the performance of several pattern recognition algorithms during dynamic contractions.

**Methods:**

A 9 class experiment was designed involving both static and dynamic situations. The performance of various feature extraction methods and classifiers was evaluated in terms of classification accuracy.

**Results:**

It is shown that, combined with a threshold to detect the onset of the contraction, current pattern recognition algorithms used on static conditions provide relatively high classification accuracy also on dynamic situations. Moreover, the performance of the pattern recognition algorithms tested significantly improved by optimizing the choice of the training set. Finally, the results also showed that rather simple approaches for classification of time domain features provide results comparable to more complex classification methods of wavelet features.

**Conclusions:**

Non-stationary surface EMG signals recorded during dynamic contractions can be accurately classified for the control of multi-function prostheses.

## Background

The myoelectric signals can be non-invasively recorded from the skin surface, and represent the electrical activity in the muscles within the detection volume of the electrodes. They are easy to acquire and have shown to be an efficient way to control powered prostheses [1]. The control strategy for multi-function prostheses widely employs the pattern-recognition approach in a supervised way. This approach assumes that different types of motion, and thus muscle activations, can be associated to distinguishable and consistent signal patterns in the surface EMG. The patterns are learned by the algorithm using some part of the data (learning process), and the algorithm is then used to predict the motions according to further data. The two main steps of pattern recognition algorithms are feature extraction and classification. First, representative features are computed from the surface EMG, and then they are assigned to classes that represent different motions. Various feature extraction methods have been explored, such as those involving time-domain features [2], variance and autoregressive coefficients [3], or time-frequency based features [4]. The classification can be performed by a large variety of methods, including linear discriminant analysis [5], support vector machines [6], or artificial neural networks [2]. With these methods, current myocontrol systems achieve >95% accuracy in a >10-class problem in intact-limbed subjects, and >85% accuracy in a 7-class problem in amputee subjects [7].

In addition to the classification approach, other methods have been developed based on pattern recognition using an estimation approach. For example, the hand kinematics can be estimated by training its association with the surface EMG of the contralateral limb with an artificial neural network [8,9]. Although this approach allows training in unilateral amputees, it not suitable for bilateral amputees who are the patient group who would most benefit from the use of active prostheses.

The limitations of the current EMG pattern recognition algorithms, which are mainly poor reliability and need for long training, prevent them from being used in clinical situations, in which the signals are not conditioned as well as in research laboratories. One of those limitations is related to the fact that current classification algorithms for EMG pattern recognition are mostly tested on stationary or transient scenarios separately. Transient surface EMG have been accurately classified using the transition as a whole[2], and stationary situations (isometric contractions) have been extensively investigated in the past decades, showing promising classification results [7,10,11]. However, these two situations have been always investigated separated, without the analysis of performance of an approach of classification of both types of signals concurrently. Therefore, this study investigates the performance of several pattern recognition classification algorithms for surface EMG signal classification, as used on static situations, when they are applied to dynamic situations, involving both static and dynamic contractions. Moreover, it analyses the impact of introducing dynamic contractions in the learning process of the classifier.

## Methods

### Subjects

Eight able-bodied subjects (5 males, 3 females; age, mean ± SD, 25.3 ± 4.6 yrs) participated in the experiment. All subjects gave their informed consent before participation and the procedures were approved by the local ethics committee.

### Procedures

**Figure 1:**
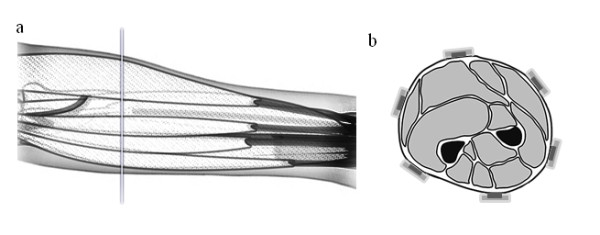
**Electrode positions**. Schematic views of the position of the electrodes: (a) lateral, (b) transversal.

The experimental protocol focused on a 9-class problem involving hand and wrist motions designed for trans-radial prostheses. The 9 classes were: wrist flexion, wrist extension, forearm supination, forearm pronation, thumb close, 4-finger close, making a fist, fingers spread open, and no motion (relax). Six pairs of Ag/AgCl surface electrodes (Ambu^® ^Neuroline 720 01-K/12, Ambu A/S, Denmark) were mounted around the dominant forearm at equal distances from each other, one third distal from the elbow joint (Figure 1). The surface EMG data were recorded in bipolar derivations, amplified with a gain of 2000 (EMG-16, OT Bioelectronica, Italy), filtered between 47 and 440 Hz, and sampled at 1024 Hz. The reference electrode was placed on the non-dominant forearm. In each experimental session, the subject was instructed to perform the 9 classes of motion twice, in random order. Each contraction was 10 s in duration, with 3 s resting periods between consecutive contractions. Each subject performed three sessions on the same day, with 5-min breaks between the sessions to minimize fatigue. The rest periods between contractions and sessions were determined according to pilot tests and subjective evaluation of the subjects on the fatigue level. In total, 54 contractions (6 per class) were performed by each subject. In each contraction, the subject was instructed to start from the rest position, to reach the target position in 3 s, to maintain the target position for 4 s, and to return to the rest position in 3 s. Thus, in each contraction, one segment of static portion (4 s in the middle), and two segments of dynamic (anisotonic and anisometric, representing the two main dynamic situations in real movements) portion (3 s at each end) were obtained. These dynamic portions contained the full path between the rest and the target position. No feedback was provided to the subjects to regulate the position, but visual validation of the motions was performed by the experimenter. A user interface was used to provide the subject with the necessary visual prompt.

### Signal analysis

The extracted data were segmented in windows of 128 samples, corresponding to 125 ms, with an overlap of 96 samples between two consecutive windows (32 samples delay between two consecutive windows) and classification was performed for each window. A sampling window of 125 ms with a delay of 30 ms has been shown to be a good trade-off between decision delay and accuracy using the majority vote [12]. The final decision was taken by majority vote on the most recent 6 results. The response time is the sum of the length of the data used to take the decision (approximately 280 ms) and the computational time (evaluated between 5 ms and 20 ms using a workstation based on an INTEL I7 860 processor). These choices make the response time in this study acceptable for prosthetic devices, as it is generally assumed that a delay shorter than 300 ms is acceptable for myoelectric control [13]. For each subject, the signal processing algorithms (see below) were tested using a three-fold cross-validation procedure. Two of the three data sets were used as learning data and the remaining data set as testing data, thus the training was done on 36 contractions (4 contractions per class) [6].

A linear discriminant analysis classifier (LDA) and two modes of Support Vector Machine (SVM) classifier with Gaussian kernel based boundary were tested. LDA was chosen because it is a simple statistical approach without any parameters to adjust, and has been shown to be one of the best classifiers for myoelectric control under stationary conditions [10]. The SVM offers a more complex approach. Depending of the choices of the kernel and parameters, SVM can generate a boundary able to follow more accurately the trends in the feature space on dynamic situations. Although the linear kernel was tested on pilot data, its parameter optimization was very specific to the training data set, resulting in poor classification accuracy. On the other hand, non-linear boundaries showed better performance. The Gaussian kernel was used, as it does not depend on a dimension selection, but on a regularization parameter, allowing to create a boundary following the trends in the feature space without creating a number of small boundaries around the outliers. The Gaussian kernel depends on two parameters for the definition of the boundary. The first mode of SVM used the One Versus Rest (OVR) approach, which separates each class with respect to all the others together, and the final decision is obtained by selecting the class maximizing the discriminant function. The second mode of SVM classifier used the One Versus One (OVO) method, which provides a decision for each pair of classes, and the final decision is obtained by majority vote. Each classifier was trained using learning sets of features extracted by one of two methods: Time Domain features and Auto Regressive coefficients (TD+AR) (as in [10]), which are simple features extracted from the signal, and the marginals of the Wavelet Transform coefficients (WT) (as in [14]). In preliminary studies, the Coiflet wavelet of order 4 has shown the best results amongst the different orders of Daubechies, Coiflet and Symmlet wavelets, and thus it was selected as the mother wavelet in the current study [15]. As for the classifiers, those two feature extraction methods were selected to compare a rather simple method (TD+AR), with a more advanced method (WT). Both methods have been successfully applied for myoelectric control in static conditions [10,14].

**Figure 2:**
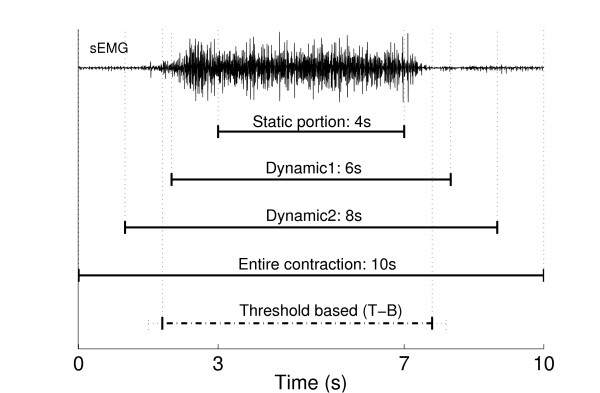
**Training intervals**. Intervals used to train the classifier displayed for one contraction along with one channel of surface EMG.

Each classifier was trained using five intervals of the contractions to study the impact of the training data selection as displayed in Figure 2. Four different intervals (sections) were obtained from the middle of each contraction as follows: 4 s (only the static portion), 6 s (the static portion and an extra 1 s at each end; Dynamic1 in Figure 2), 8 s (the static portion and an extra 2 s at each end; Dynamic2 in Figure 2) and 10 s (the entire contraction). Finally, an additional training section was threshold-based (T-B, see below for description of the threshold algorithm), so that the current window was used for training only if its EMG activity exceeded the threshold.

A threshold was applied to each window, comparing the activity in the multi-channel surface EMG to a reference level taken during the rest. The Teager-Kaiser energy operator [16] was used to detect the onset of the contractions. For each window, an activity value was given to each channel using the Teager-Kaiser operator. This value was thresholded by a coefficient multiplied by the values obtained at rest. The window was considered as active if at least one channel crossed the threshold. For each subject, the coefficient of the threshold was determined on the static portions from the learning data. Its value was maximized under the constraints to have more than 97% of the windows from all classes active, and no less than 85% of the windows from each individual class active. These two conditions were determined on pilot data and have shown to be consistent across the subjects. The threshold for each subject was obtained only from the learning data. The threshold values were rather different between subjects and channels, spanning two orders of magnitude, mainly because of the difference in electrode placement and background noise. The level of normalized EMG activity during the contractions varied between 56% and 92% depending on the class.

The cross-validation procedure was applied to each combination of feature set, training section and classifier. The accuracy was evaluated on the testing set on all classes (including the rest class). The classification action was performed if the EMG activity in the current window exceeded the threshold obtained from the training set. Otherwise the current window was considered as belonging to the rest class.

## Results

**Figure 3:**
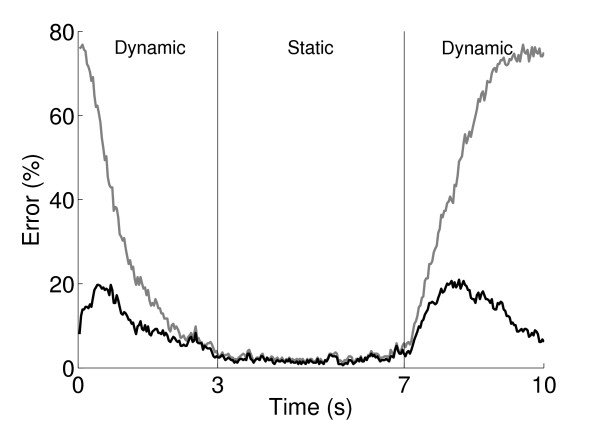
**Errors position**. Position in time of classification errors during contractions, with threshold (black) and without threshold (grey). For each window position, the error is expressed as a percentage, averaged across subjects and contractions on that position.

Various pattern recognition methods are capable of high performance in myoelectric control under static conditions [11], which was confirmed by a preliminary analysis of the data in this study. As shown in Figure 3 without using the threshold, most of the classification errors were clustered at the beginning and end of the contractions, when the subject was near the rest position. Applying the threshold substantially improved the performance by reducing the confusion of the rest class with other classes.

**Figure 4:**
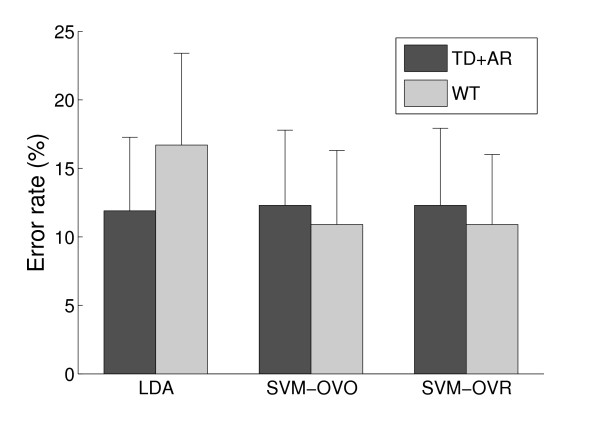
**Error rates on static training**. Error rate (mean and standard deviation) of the combinations feature set and classifier when training on the static part.

Figure 4 displays the error rate of each pair of feature set and classifier when the training was exclusively performed on the static part of the contractions. Using this training set, when combined with a threshold, a simple LDA classifier with a TD+AR feature set achieved, on average, more than 88% accuracy in dynamic situations. The use of a more complex classifier (SVM-OVR) and feature set (WT) slightly improved the performance (~1% increase in accuracy). Figure 4 also indicates that the LDA classifier is more compatible with the TD+AR feature set than with the WT feature set. Indeed, the use of the marginals, which is a non linear operator, reduces the compatibility with the linear nature of the LDA.

**Figure 5:**
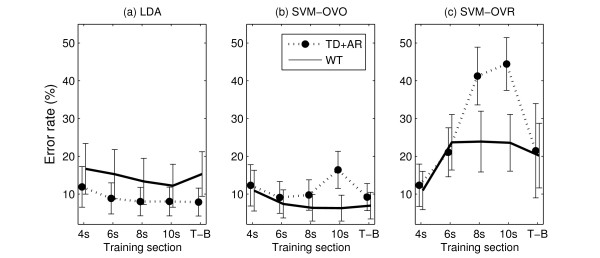
**Error rates depending on the training section**. Performance (mean and standard deviation) of the different combinations of feature sets and classifiers (a): LDA; (b): SVM-OVO; (c): SVM-OVR, depending on the training sections as defined in Figure 2.

Figure 5(a) confirms that LDA does not perform optimally with the WT feature set. In addition, it shows that the combination of LDA with TD+AR features determines high performance (error limited to ~8%) when trained using some part of the dynamic portion in addition to the static portion. Although the differences in performance when using different dynamic sections (sections including a portion of the dynamic contraction) for training were very low (<0.6%), the best results were obtained using the threshold based training section, which provides automatically an efficient way to determine which portion of the signals should be used as the training set.

Figure 5(b) shows that the SVM-OVO classifier with WT features determines high performance when including the dynamic portions in the training set. An error rate of 6.3% was reached when using the entire contraction as training section. When using the TD+AR feature set, the performance also increased when using the dynamic portions for training and reached a 9.7% error when using the 8-s training section. Figure 5(c) indicates that the performance of the SVM-OVR classifier deteriorates when more dynamic data are included in the training set. The OVR mode for SVM creates a boundary for each class separating it from all the others. Including the dynamic portion in the training set increases substantially the number of windows available for each class, and so the unbalance between the sizes of the two classes during the learning process increases. This reduces the efficiency of the SVM learning algorithm, which results in poorly generated boundaries.

A three way ANOVA was applied on the error rate with the algorithm (TD+AR/LDA or WT/SVM-OVO) and the training section (5 training sections) as the factors and the subject considered as a random variable. Only the TD+AR/LDA and WT/SVM-OVO were investigated with this analysis since they are the most relevant combinations, as shown above. The analysis of the results revealed a significant effect from both factors and from the interaction between them (P < 0.005).

**Figure 6:**
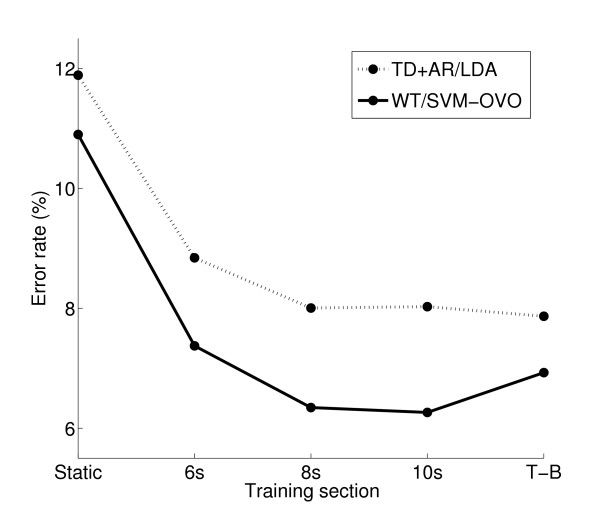
**Analysis of variance-Interaction**. Error rates of the two algorithms included in the ANOVA depending on the training sections.

Figure 6 represents the significance of the interaction between the algorithm and the training section. A Sheffe post hoc test was applied to the training section factor, for both algorithms separately, to reveal the significance levels amongst pairs of training sections. For both algorithms, the static training section (4 s) showed significantly higher error rate than all the other training modalities investigated. However, the 6 s, 8 s, 10 s and T-B training sections did not provide significantly different results for any of the two algorithms.

Although the previous results show a significant improvement using the dynamic portions for training, the inter-subject variability obscures the relative performance across the different training sections. This variability is related to two main factors:

• subjects' ability to perform the exact movement following a cue,

• efficacy of the threshold on the resulting surface EMG.

**Figure 7:**
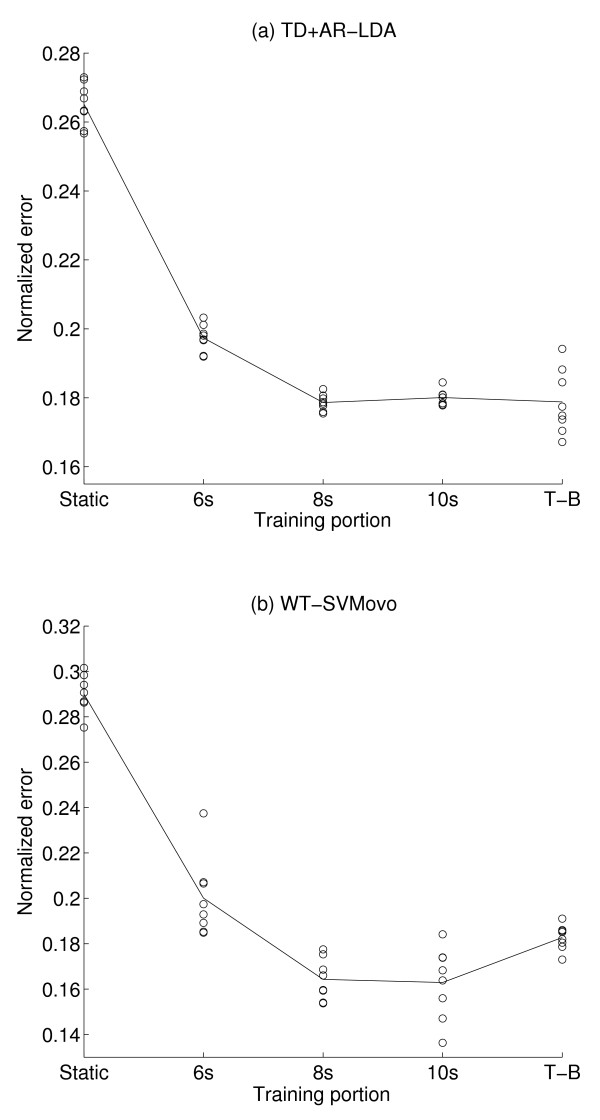
**Normalized errors**. The normalized errors depending on the training section for the TD+AR/LDA algorithm (a) and the WT/SVM-OVO (b).

Therefore, we further define ∑*_i _*, an index that provides a measure of the overall "ability" on the subject *i *[15]:

Where each  is the error rate for the subject *i *using the training section with a length of *x *(T is for Threshold-based). We then normalize the error for each training section with respect to the overall index of ability for each subject:

These normalized errors reveal the relative performances of the training sections, and allow the results for each subject to be displayed on the same scale. Figure 7 depicts the mean across subjects of the normalized errors for each training section, as well as the results for each subject. The relative performance of the training sections confirmed the trend of the non-normalized error observed in Figure 4, and the individual representations are in most cases well clustered around the mean for each training section.

A one way ANOVA was applied on the normalized errors for each algorithm using the training section as factor. In both cases, the results confirmed that the effect of the training section was significant. A Sheffe post hoc test was applied on these results and confirmed the previous results for the TD+AR/LDA algorithm. For the WT/SVM-OVO algorithm, the post hoc test revealed significant differences between the training sections, dividing them in three groups (section 8 s and 10 s; section 6 s and T-B; Static section). Table 1 summarize all results.

**Table 1:** Results summary

	LDA		SVM ovo		SVM ovr	
**Training Data sections**	**TD**	**WT**	**TD**	**WT**	**TD**	**WT**

Stationary: 4 s	11.9 ± 5.38	16.7 ± 6.72	12.3± 5.47	10.9 ± 5.41	12.3 ± 5.61	10.9 ± 5.09

Dynamic 1: 6 s	8.84 ± 4.13	15.3 ± 6.53	9.10 ± 4.22	7.37 ± 3.72	21.1 ± 6.49	23.7 ± 7.35

Dynamic 2: 8 s	8.00 ± 3.79	13.3 ± 6.11	9.75 ± 4.03	6.34 ± 3.53	41.3 ± 7.65	23.9 ± 8.06

All 10 s	8.03 ± 3.82	12.2 ± 5.70	16.4 ± 4.92	6.26 ± 3.44	44.4 ± 7.00	23.6 ± 7.51

Threshold	7.87 ± 3.70	15.3 ± 5.91	9.19 ± 3.58	6.93 ± 3.55	21.5 ± 12.5	20.2 ± 8.55

Summary of the results, with the average error rate across all the subjects depending on the feature extraction method, the classifier, and the training section.

## Discussion

The results of the study show that, using a threshold to detect the onset of the motion, surface EMG during dynamic tasks can be classified with accuracy comparable to that obtained in static situations, when the training section is properly selected (Table 1).

Including some dynamic portions (6 s, 8 s, 10 s, T-B) of sEMG during the learning process significantly improved the performance of both LDA and SVM based algorithms compared to the static training (4 s). The inferior performance of the SVM-OVR classifier when dynamic portions are included in the training set is not likely related to the inclusion of the dynamic part. Rather, it is more likely due to the unbalance of size during the learning process, i.e. a 1 to 8 ratio between one class compared to all the others together. Reducing the number of samples taken for the elements of the biggest class during learning could solve this issue, but would require an additional step, and an optimization of the samples to select, which is beyond the scope of this study.

Although the best results were obtained using the pair WT/SVM-OVO (6.3% ± 3.3% error), the disadvantage of this combination is the relatively high requirement in terms of optimization. Indeed, the SVM requests at least one penalization parameter, and in case of non-linear boundary two parameters which must be optimized. In addition, this study shows that the optimization of the training section has a great impact on the performance. Unfortunately, the effect of these factors seemed to have interaction, thus they have to be optimized together. This increases significantly the time required to train the algorithm and the amount of data required for training.

On the other hand, the combination TD+AR/LDA showed a good performance (8.0% ± 3.5% error), and it does not require any optimization. Moreover, this study showed that this combination is much less sensitive to the training section compared to the WT/SVM-OVO combination, and that it reaches its optimal performance if some dynamic portions are included in the learning process. This shows that the selection of the training section in that case can be done automatically, by taking the entire contraction as training, or by using a threshold in activation. This results in a completely automated algorithm, that can be trained within a short period of time, and adapted to each patient using the threshold selection. Therefore, this combination is more suitable for clinical applications in which the training must be kept as short as possible. Interestingly, this combination of features and classifier has also shown to be the best suitable real-time myoelectric classification algorithm under static conditions [12].

In addition to the focus on classification, this study also presents a method for movement onset detection. The results presented depend on the accuracy of this method. The threshold was adapted individually, and applied identically for each investigated algorithm. Therefore, the impact of threshold selection on the relative performances of these algorithms is minimal. This approach aimed to simulate the clinical situations (i.e., one or more fixed thresholds per recording site) so that results obtained are as consistent as possible with what one would expect in real applications. The main result of the current study is that the relatively simple TD+AR/LDA approach maintains relatively high performance under the dynamic conditions tested. This result was obtained on healthy subjects. Further investigations will involve amputee patients as end-users of the system. According to previous work [7], it is expected that the results of this study will translate to patients, potentially with a decrease in the overall accuracy.

Finally, it is important to notice that this study focused on the transitions between various movements and the rest position. Further optimization could be achieved by involving the transitions between all the combinations of active classes in the learning process. This would however increase the amount of training data and training time significantly making it impractical for clinical applications. Thus, a classifier less sensitive to such kind of training requirements as well as methods to decrease the retraining requirements of the algorithms should be further investigated. This remains a challenge for the ongoing studies along with proportional and simultaneous control.

## Conclusions

The dynamic portions of EMG signals are important for real myocontrol systems and thus must be included in the learning process in order to achieve an overall high classification accuracy. When the learning set is properly chosen, rather simple pattern recognition approaches provide similar classification accuracies for dynamic as for static situations.

## Competing interests

The authors declare that they have no competing interests.

## Authors' contributions

TL participated in the design of the study, carried out the experiments, analysis, and drafted the manuscript. NJ participated to the design and realization of the study and to the manuscript preparation, DF participated to the design and coordination of the study and to the manuscript preparation. All authors read and approved the final manuscript.

## References

[B1] Scott RN Parker PA Myoelectric prostheses: State of the art J Med Eng Technol 1988 12 Suppl 4 143 151 3057209 10.3109/03091908809030173

[B2] Hudgins B Parker P Scott RN A new strategy for multifunction myoelectric control IEEE Transactions on Biomedical Engineering 1993 40 Suppl 1 82 94 8468080 10.1109/10.204774

[B3] Graupe D Cline WK Functional separation of EMG signals via ARMA identification methods for prosthesis control purposes IEEE Trans Syst Man Cybern 1975 5 Suppl 2 252 259

[B4] Farry KA Fernandez JJ Abramczyk R Novy M Atkins D Applying genetic programming to control of an artificial arm Myoelectric Controls Conf.: Issues Upper Limb Prosthetics, Fredericton 1997 50 55

[B5] Huang Y Englehart KB Hudgins B Chan ADC A Gaussian mixture model based classification scheme for myoelectric control of powered upper limb prostheses IEEE Transactions on Biomedical Engineering 2005 52 Suppl 11 1801 1811 16285383 10.1109/TBME.2005.856295

[B6] Shenoy P Miller KJ Crawford B Rao RPN Online electromyographic control of a robotic prosthesis IEEE Transactions on Biomedical Engineering 2008 55 Suppl 3 1128 1135 18334405 10.1109/TBME.2007.909536

[B7] Hargrove LJ Li G Englehart KB Hudgins BS Principal components analysis preprocessing for improved classification accuracies in pattern-recognition-based myoelectric control IEEE Trans Biomed Eng 2009 56 Suppl 5 1407 1414 19473932 10.1109/TBME.2008.2008171

[B8] Muceli S Jiang N Farina D Multichannel surface EMG based estimation of bilateral hand kinematics during movements at multiple degrees of freedom IEEE-EMBC 2010 6066 6069 10.1109/IEMBS.2010.5627622 21097125

[B9] Sebelius F Eriksson L Balkenius C Laurell T Myoelectric control of a computer animated hand: A new concept based on the combined use of a tree-structured artificial neural network and a data glove J Med Eng Technol 2006 30 Suppl 1 2 10 16393847 10.1080/03091900512331332546

[B10] Hargrove L Scheme E Englehart K Hudgins B Principal components analysis tuning for improved myoelectric control 2007 10.1109/IEMBS.2007.4353851 18003517

[B11] Chu JU Moon I Lee YJ Kim SK Mun MS A supervised feature-projection-based real-time EMG pattern recognition for multifunction myoelectric hand control IEEE/ASME Transactions on Mechatronics 2007 12 Suppl 3 282 290

[B12] Englehart K Hudgins B A robust, real-time control scheme for multifunction myoelectric control IEEE Transactions on Biomedical Engineering 2003 50 Suppl 7 848 854 12848352 10.1109/TBME.2003.813539

[B13] Farrell TR Weir RF The optimal controller delay for myoelectric prostheses IEEE Transactions on Neural Systems and Rehabilitation Engineering 2007 15 Suppl 1 111 118 17436883 10.1109/TNSRE.2007.891391 PMC8173529

[B14] Lucas MF Gaufriau A Pascual S Doncarli C Farina D Multi-channel surface EMG classification using support vector machines and signal-based wavelet optimization Biomedical Signal Processing and Control 2008 3 Suppl 2 169 174

[B15] Englehart K Hudgins B Parker PA Stevenson M Classification of the myoelectric signal using time-frequency based representations Medical Engineering and Physics 1999 21 Suppl 6-7 431 438 10624739 10.1016/s1350-4533(99)00066-1

[B16] Solnik S DeVita P Rider P Long B Hortobágyi T Teager-Kaiser Operator improves the accuracy of EMG onset detection independent of signal-to-noise ratio Acta of bioengineering and biomechanics/Wroclaw University of Technology 2008 10 Suppl 2 65 19032000 PMC2596643

